# Quantitative assessment of preoperative serum thyrotropin level and thyroid cancer

**DOI:** 10.18632/oncotarget.9201

**Published:** 2016-05-06

**Authors:** Jiaojiao Zheng, Chen Li, Weihui Lu, Cong Wang, Zhilong Ai

**Affiliations:** ^1^ Department of General Surgery, Zhongshan Hospital, Fudan University, Shanghai 200032, P.R. China; ^2^ Department of General Surgery, Zhongshan Hospital (Qingpu Branch), Fudan University, Shanghai 201700, P.R. China

**Keywords:** thyroid stimulating hormone, thyroid cancer, TNM staging, lymph node metastasis, meta-analysis

## Abstract

Thyroid stimulating hormone (TSH) is the major growth factor for thyrocytes, but the pathogenic role of serum TSH in thyroid cancer (TC) is unknown. The association between TSH level and the development of thyroid cancer has been widely evaluated recently. However, the results remain conflicting. To develop an understanding of the relationship between TSH exposure and thyroid cancer, a meta-analysis of 56 studies involving 20227 thyroid cancer cases and 50003 controls with benign thyroid nodule was performed. Overall, significantly increased TSH level was observed in thyroid cancer patients compared with controls (RoM: 1.44, 95% CI: 1.32–1.56, *P* < 10^−5^). The pooled analyses also revealed that higher serum TSH level were significantly associated with the size of TC nodule and malignancy as well as lymph node metastasis. Furthermore, significantly increased THS levels were observed preferentially for papillary thyroid cancer when stratified by histological type of tumors. However, the diagnostic value of TSH level for TC might be limited. These results suggest that higher serum TSH concentration is associated with an increased risk of thyroid cancer.

## INTRODUCTION

Thyroid cancer (TC) is the most common endocrine malignancy [1], which is classified into four main histology groups: papillary thyroid cancer (PTC), follicular thyroid cancer (FTC), medullary thyroid cancer (MTC), and undifferentiated or anaplastic thyroid carcinomas [2, 3]. Mostly, TC presents clinically as a solitary nodule or as a dominant nodule within a multinodular thyroid gland [4]. Although the vast majority of thyroid nodules detected by ultrasonography are reported to be benign [5, 6], 5% ~ 15% of clinically apparent thyroid nodules are malignant [7]. Therefore, identification malignancy in patients with thyroid nodules could be a great challenge to clinicians.

Thyroid stimulating hormone (TSH) is a glycoprotein, which stimulates secretion of thyroid hormones, maintenance of thyroid-specific gene expression, and gland growth [8, 9]. Suppression of serum TSH concentrations by administering exogenous thyroxine is a mainstay in clinical management to TC, with good evidence of mortality benefit in high-risk patients [10]. Recently, several studies have suggested that a higher serum concentration of TSH is associated with thyroid malignancy in patients with thyroid nodules [11–13]. Despite the biological plausibility of TSH as a risk factor of TC, existing results remain inconclusive and inconsistent. The lack of concordance reflects limitation in these studies, such as ethnic difference, phenotypic heterogeneity, limited statistical power and bias in the study design. We therefore performed a meta-analysis on clinical studies to develop an understanding of the relationship between serum TSH level and TC.

## RESULTS

### Characteristics of the included studies

**Figure 1:**
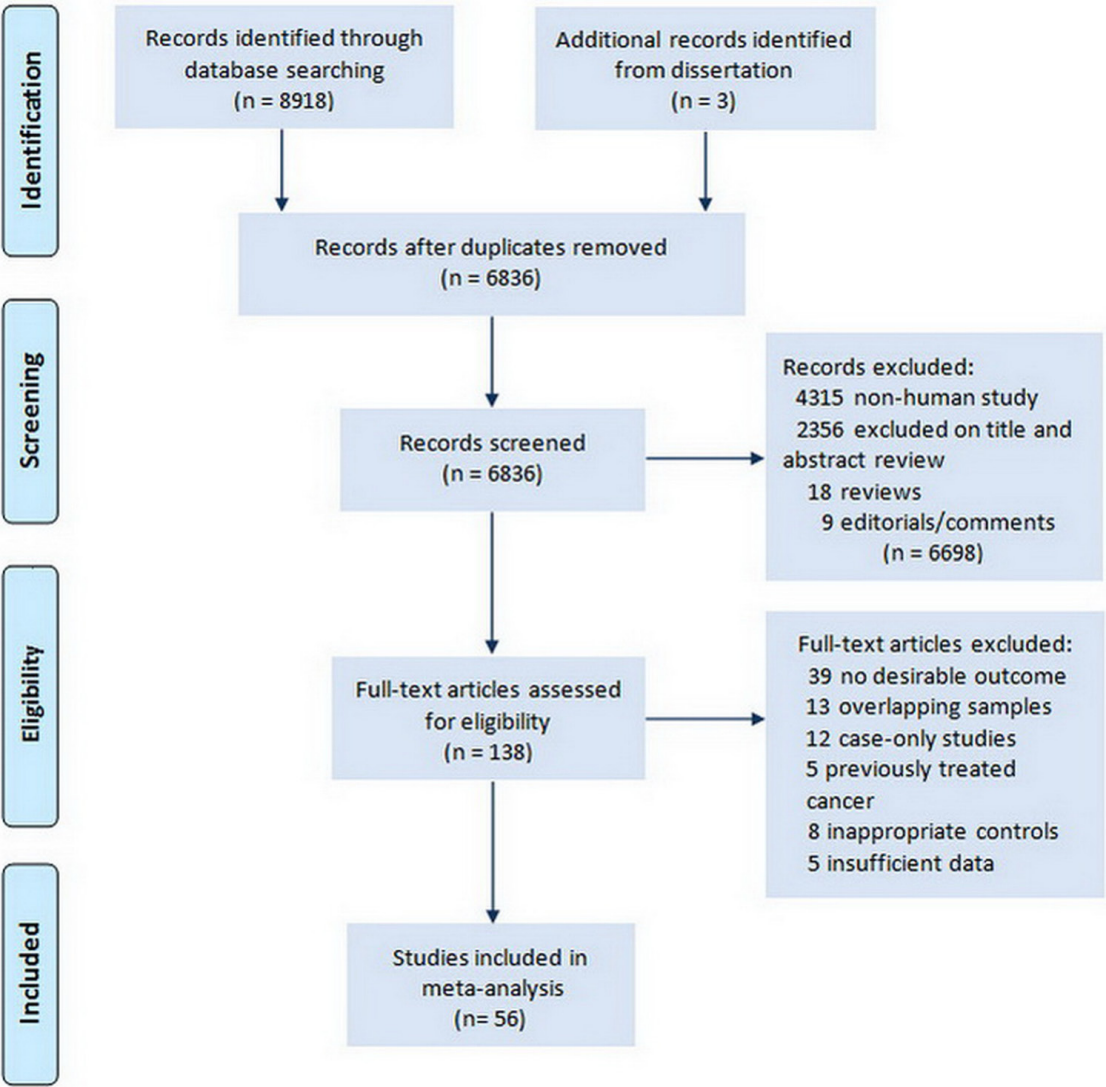
Flow chart of literature search for studies examining serum TSH level and thyroid cancer

Our primary search generated 8,918 citations. After duplicates were removed 5484 citations remained for title and abstract screening. Results from the literature search and study selection process were shown in Figure 1. Finally, 56 studies [11, 14–65] and 3 unpublished data with 70230 subjects in total, including 20227 cases of thyroid cancer were included in the meta-analysis; most were retrospective cross-sectional studies. Characteristics of these studies summarized in Supplementary Table 1.

### TSH levels between TC patients and controls

**Figure 2:**
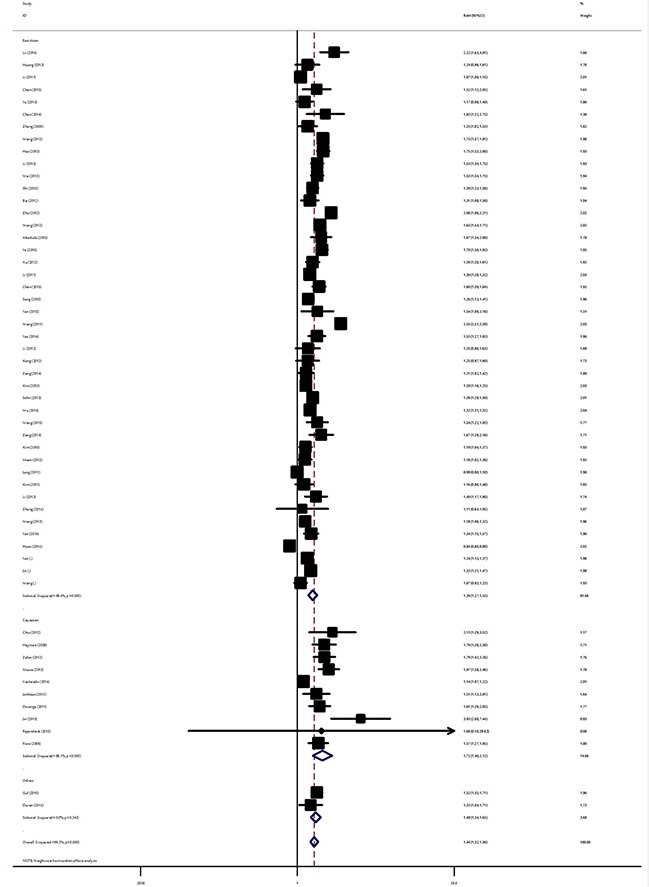
Ratio of the mean (RoM) serum TSH levels in TC patients compared to the controls and the 95% confidence intervals, as stratified by ethnicity

Table 1 summarize the main results of the meta-analysis for TSH level and TC. Overall, the random effects model summarising all 56 comparisons revealed that TC patients had significantly higher mean TSH levels compared to controls (RoM: 1.44, 95% CI: 1.32–1.56, *P* < 10^−5^). In different ethnicities, the pooled RoM for East Asian TC patients was 1.39 (95% CI: 1.27–1.53, *P* < 10^−5^), 1.72 for Caucasian cases (95% CI: 1.40–2.12, *P* < 10^−5^) and 1.49 for the other ethnic populations (95% CI: 1.33–1.65, *P* < 10^−5^; Figure 2). As for studies with small and moderate sample size, the summary RoM of serum TSH level for TC was 1.54 (95% CI: 1.42–1.67, *P* < 10^−5^) and 1.33 (95% CI 1.20–1.47, *P* < 10^−5^) in studies with small and moderate sample size, respectively. When analysis was restricted to the 12 studies with at least 500 TC cases, which should be less prone to selective publication than smaller studies, yielded an pooled RoM of 1.33 (95% CI: 1.05–1.68, *P* = 0.017). Paediatric thyroid cancer appears to be unique and quite different from the adult form. Significant increased serum TSH levels were both found in adults cases (RoM: 1.42, 95% CI: 1.31–1.55, *P* < 10^−5^) and paediatric TC patients (RoM: 1.99, 95% CI: 1.63–2.44, *P* < 10^−5^). In the stratified analysis by study design, a significant increased TSH was detected among studies using retrospective cross-section design and studies using other design (Table 1). Not all researchers reported data in the same way, and most articles reported results for TSH using mean and standard deviation. Analysis restricted to the 49 studies with sufficient data, which should be less prone to data bias from estimations of mean and SD, yielded an overall RoM of 1.44 (95% CI: 1.31–1.57, *P* < 10^−5^; Supplementary Figure 1).

**Table 1:** Main results of overall and subgroups analysis for serum TSH level and TC

Overall and subgroups analyses	No. of studies	No. of cases/controls	RoM (95% CI)	*P* (Z)	*P* (Q)	*I*^2^ (%)
All	56	20227/50003	1.44 (1.32–1.56)	< 10^−5^	< 10^−5^	99.2
Ethnicity						
Asian	44	18291/35339	1.39 (1.27–1.53)	< 10^−5^	< 10^−5^	99.4
Caucasian	10	1660/11827	1.72 (1.40–2.12)	< 10^−5^	< 10^−5^	86.1
Others	2	276/2837	1.49 (1.33–1.65)	< 10^−5^	0.34	0
Age						
Adult	53	20151/49794	1.42 (1.31–1.55)	< 10^−5^	< 10^−5^	99.3
Children	3	76/209	1.99 (1.63–2.44)	< 10^−5^	0.96	0
Sample size						
Small (No. cases < 200)	29	2605/6116	1.54 (1.42–1.67)	< 10^−5^	< 10^−5^	91.8
Moderate (No. cases between 200–500)	15	4922/12356	1.33 (1.20–1.47)	< 10^−5^	< 10^−5^	89.8
Large (No. cases > 500)	12	12700/31531	1.33 (1.05–1.68)	0.017	< 10^−5^	99.8
Study design						
Retrospective cross-sectional study	49	13834/38454	1.43 (1.31–1.56)	< 10^−5^	< 10^−5^	99.3
Others	7	6393/11549	1.52 (1.24–1.85)	< 10^−5^	< 10^−5^	74.0

### TSH effects on size, stage, metastasis and histological subtype of TC

The data on TSH level of TC patients stratified by tumour size, TNM stages and lymph node metastasis status were available in 8, 7, and 10 studies respectively (Table 2). When tumour sizes were classified into subgroups defined as < 1 cm and > 1 cm, analysis demonstrated that patients with larger tumour size had significantly increased TSH levels (RoM = 1.56, 95% CI: 1.22–2.00 vs. RoM = 1.22, 95% CI: 1.10–1.36; *P* = 0.01) compared with patients with smaller one (Table 1). The patients with advanced disease (stage III and IV) had a significantly higher mean TSH relative to those with stage I and II disease. The RoM TSH of those with advanced disease was 2.09 (95% CI: 1.60–2.72) vs. 1.34 (95% CI: 1.17–1.54) (*P* < 10^−5^). There was significant difference in TSH levels between TC cases with and without lymph node metastasis (RoM: 2.32 vs. 1.46, *P* = 0.001). Given the biological differences between the histological types of TC, subgroup analyses by histological types found that serum TSH levels were significantly increased among PTC (*P* < 10^−5^). However, no significant differences in TSH levels were detected in FTC (*P* = 0.95).

**Table 2:** TSH level and tumor size, TNM stage, lymph node metastasis

Subgroups	No. of studies	No. of cases/controls	RoM (95% CI)	*P* (Z)	*P* (Q)	*I*^2^ (%)
Tumor size						
> 1 cm	8	2262/6156	1.56 (1.22–2.00)	< 10^−4^	< 10^−5^	97.4
< 1 cm	8	2481/6156	1.22 (1.10–1.36)	< 10^−4^	< 10^−5^	85.3
TNM stage						
I and II	7	2335/13126	1.34 (1.17–1.54)	< 10^−5^	< 10^−5^	96.7
III and IV	7	903/13126	2.09 (1.60–2.72)	< 10^−5^	< 10^−5^	98.5
Lymph node metastasis						
No	10	2021/10975	1.46 (1.23–1.73)	< 10^−5^	< 10^−5^	96.9
Yes	10	631/10975	2.32 (1.79–3.02)	< 10^−5^	< 10^−5^	97.6
Histological type						
PTC	5	2978/1786	1.27 (1.17–1.38)	< 10^−5^	< 10^−5^	81.1
FTC	5	87/1786	0.99 (0.73–1.34)	0.95	< 10^−5^	81.4

### Association between TSH levels and TC Risk

Thyroid functional autonomy, defined as serum TSH levels below the lower limit of the normal range (0.4 mU/ml), was used as reference group and a TSH of 4.2 or greater was considered above the upper end of normal TSH [66]. The risk of higher serum TSH for TC was significantly increased with OR of 7.25 (95% CI: 4.22–12.47, *P* < 10^−5^); while the OR for TC with TSH between 1.35 and 2.3 was 1.84 (95% CI: 1.27–2.66, *P* = 0.001; Supplementary Figure 2).

### The diagnostic value of TSH for TC

**Figure 3:**
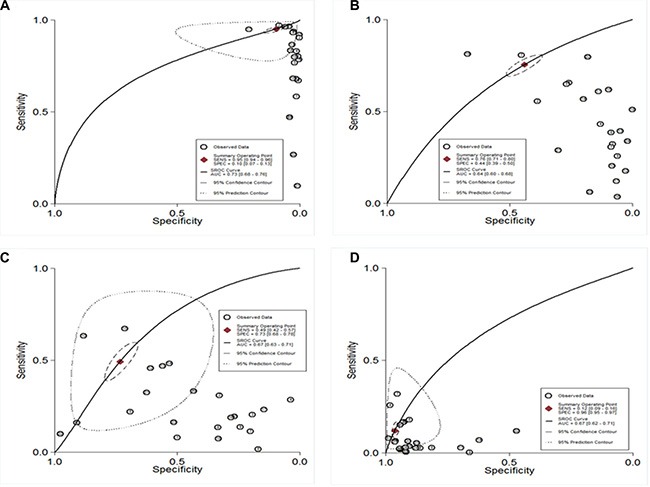
SROC curve of diagnostic meta-analysis (**A**) TSH cut-off < 0.5 mU/L; (**B**) TSH cut-off 0.5–1.5 mU/L; (**C**) TSH cut-off 1.5–2.5 mU/L; (**D**) TSH cut-off > 3.5 mU/L).

**Figure 4:**
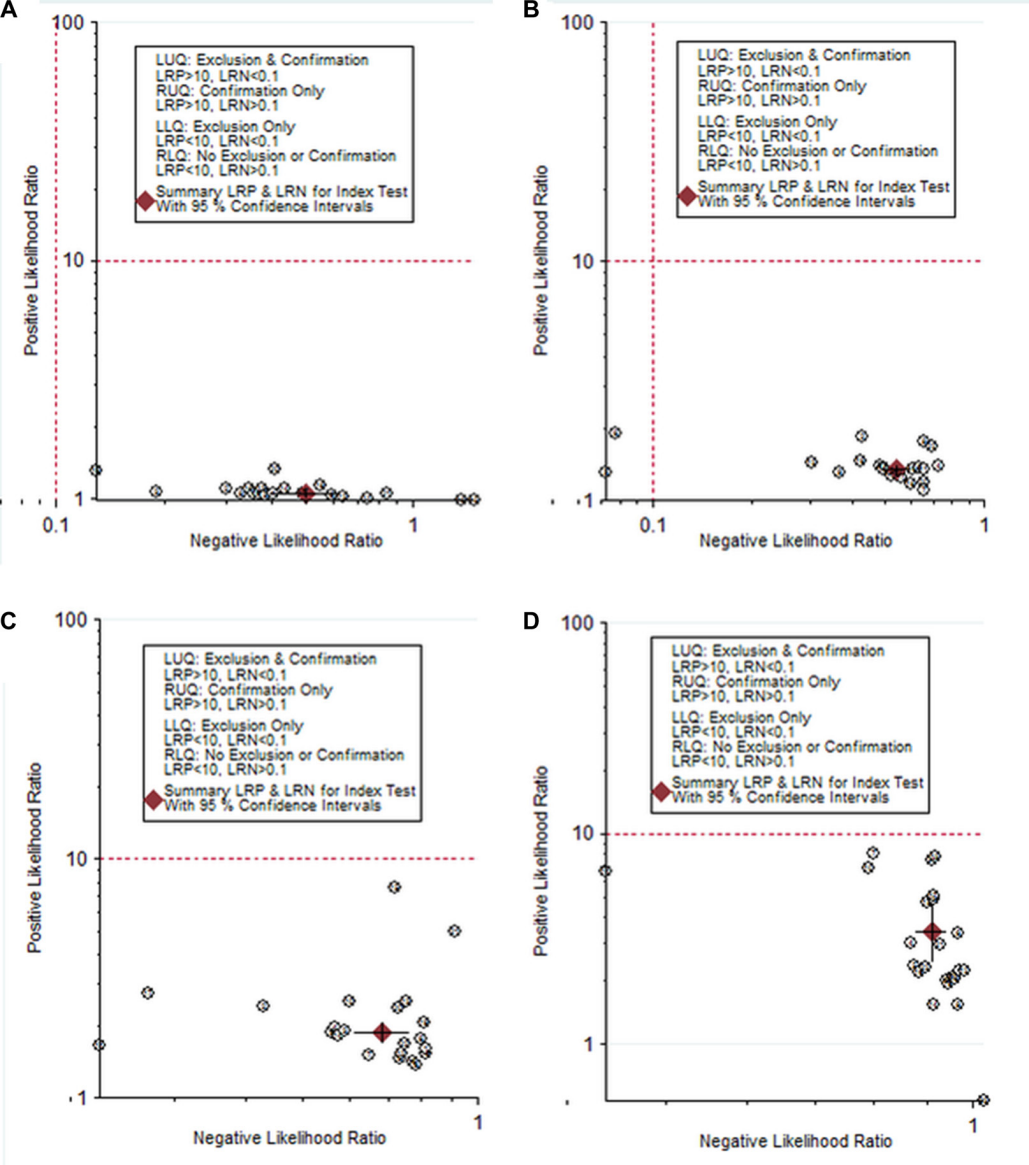
The likelihood ratio matrix of TSH for the diagnosis of thyroid cancer (**A**) TSH cut-off < 0.5 mU/L; (**B**) TSH cut-off 0.5–1.5 mU/L; (**C**) TSH cut-off 1.5–2.5 mU/L; (**D**) TSH cut-off > 3.5 mU/L).

To investigate the diagnostic value of TSH for thyroid cancer, a diagnostic meta-analysis was conducted. Many studies showed the distributions of cases and controls according to the TSH range. To explore the optimal cut-off value, true positives, false positives, false negatives, and true negatives for each individual study were calculated according to different cut-off value. The pooled specificity, sensitivity and area under the SROC were summarized in Figure 3. The pooled specificity was high (0.96, 95% CI: 0.95–0.97) when the cut-off value was set as larger than 3.5 mU/L, while the sensitivity was poor (0.12, 95% CI: 0.09–0.16). When the cut-off value was less than 0.5 mU/L, the pooled sensitivity was high (0.95, 95% CI: 0.94–0.96) and the pooled specificity was poor (0.10, 95% CI: 0.07–0.13). Subgroup analysis stratified by ethnicity showed there was no significant difference between Asians and Caucasians (Table 3). Likelihood ratios were used to evaluate clinical utility of the diagnostic test. A clinically useful test was defined with a PLR > 10 and a NLR < 0.1. In our meta-analysis, no cut-off value can reach the threshold of clinically useful test (Figure 4).

**Table 3:** Summary of diagnostic meta-analysis of TSH for thyroid cancer

Cut-off of TSH	Ethnicity	*n*	Specificity (95% CI)	Sensitivity (95% CI)	AUSROC curve (95% CI)
< 0.5	All	21	0.10 (0.07–0.13)	0.95 (0.94–0.96)	0.73 (0.68–0.76)
	Asian	15	0.08 (0.06–0.10)	0.96 (0.95–0.97)	0.80 (0.76–0.83)
	Caucasian	6	0.13 (0.09–0.17)	0.90 (0.88–0.92)	0.70 (0.66–0.74)
0.5–1.5	All	22	0.44 (0.39–0.50)	0.76 (0.71–0.80)	0.64 (0.60–0.68)
	Asian	11	0.41 (0.37–0.46)	0.78 (0.73–0.81)	0.63 (0.59–0.67)
	Caucasian	11	0.44 (0.60–0.69)	0.76 (0.68–0.83)	0.65 (0.60–0.69)
1.5–2.5	All	22	0.73 (0.68–0.78)	0.49 (0.42–0.57)	0.67 (0.63–0.71)
	Asian	15	0.71 (0.64–0.72)	0.55 (0.46–0.63)	0.69 (0.64–0.72)
	Caucasian	7	0.80 (0.68–0.88)	0.36 (0.27–0.47)	0.58 (0.54–0.63)
> 3.5	All	24	0.96 (0.95–0.97)	0.12 (0.09–0.16)	0.68 (0.64–0.72)
	Asian	18	0.97 (0.95–0.98)	0.13 (0.09–0.18)	0.71 (0.67–0.75)
	Caucasian	6	0.96 (0.94–0.97)	0.10 (0.08–0.13)	0.47 (0.42–0.51)

### Heterogeneity exploration

Significant heterogeneity was present among the included studies in overall and subgroup analysis (*P* < 0.05). As the formal test for heterogeneity may not be powerful enough, meta-regression was used to evaluate potential effect modifiers by including ethnicity, sample size, study design, mean age, sex distribution and study quality as covariates. In meta-regression analysis, it was found that sample size (coefficient = −0.09, *P* < 10^−5^) and ethnic population (coefficient = 0.08, *P* = 0.004) was potential source of heterogeneity; while study design (*P* = 0.90), mean age of cases (*P* = 0.18) and controls (*P* = 0.79), sex distribution among cases (*P* = 0.09) and controls (*P* = 0.76), and study quality (*P* = 0.05), did not significantly explain between-study heterogeneity. Furthermore, Galbraith plot analyses of all included studies were used to assess the potential sources of heterogeneity. Five studies were found to be the contributors of heterogeneity (Supplementary Figure 3).

### Sensitivity analyses and small study effect

To assess the extent to which individual studies with extremely large RoMs influenced the summary RoM, one-way sensitivity analyses was conducted. The exclusion of the study by Jin et al. [56] that included the largest RoM estimate reduced between-study heterogeneity but did not appreciably change the summary RoM (1.43; 95% CI: 1.28–1.59; P_heterogeneity_ < 10^−5^, *I*^2^ = 99.0%). One recent study [58] with the smallest number of cases (*n* = 14) yielded large variance in the effect estimate; removing this study led to almost the same summary RoM (1.44; 95% CI: 1.32–1.56; P_heterogeneity_ < 10^−5^, *I*^2^ = 99.3%). However, omitting the study by Wang et al. (14), which included large sample size and heterogeneous results, did substantially influence the summary RoM (1.39; 95% CI: 1.33–1.45; P_heterogeneity_ < 10^−5^, *I*^2^ = 93.5%). Sensitivity analysis indicated that the results of this meta-analysis were stable, with RoMs and 95% CIs ranging from 1.39 (95% CI: 1.33–1.45, *P* < 10^−5^) to 1.45 (95% CI: 1.30–1.61, *P* < 10^−5^) (Supplementary Figure 4).

We found no evidence of small study effects (*P* = 0.39 by Egger regression test; Supplementary Figure 5). The shape of the funnel plots was symmetrical (*P* = 0.06 Begg adjusted rank correlation test, Supplementary Figure 6).

## DISCUSSION

This is the first meta-analysis addressing serum TSH and TC risk, and our results indicate that TC patients have significant increased level of serum TSH, supporting recommendations of TSH suppressive therapy to reduce the risk of TC recurrence and increased survival [67]. Significant associations were observed in East Asians, Caucasians, and other ethnic populations, suggesting the importance of TSH as a predictor of the risk of TC in different ethnicity with different genetic backgrounds and living environments. We also observed that the effect size in small and moderate studies was stronger than that in studies with large sample size. Indeed, small sample sized association studies lack statistical power and have resulted in apparently contradicting finding [68]. Larger studies of different ethnic populations, strict selection of patients will be required in the future.

Association between serum TSH concentrations and likelihood of differentiated thyroid carcinoma was mainly investigated in adults. However, paediatric thyroid cancer appears to be unique, and quite different from the adult form in terms of epidemiology and natural history [69]. By combining all the evidence available, we also observed a higher serum TSH concentration in children and adolescents with differentiated thyroid cancer compared with those with benign thyroid nodules. Given the limitations of pediatric studies and the small size, our results may be overinflated and large scale researches will be required to confirm our findings.

In subgroup analysis by nodules size (> 1 cm vs. < 1 cm), positive association was maintained regardless of the size of tumor. This suggests that the pathophysiology of TSH in tumorigenesis was similar among TC regardless of tumor size. Therefore, relatively high serum TSH concentrations may not be useful as a malignancy predictor in the assessment of thyroid nodules. When patients with TC were grouped according to TNM, we found that mean serum TSH levels were significantly higher in those with advanced stage disease when compared with those with more localised (stage I and II) disease. A similar finding was present in patients with lymph node metastasis with respect to those with no evidence of node metastasis. These results of advanced disease further suggest that TSH might be involved in the pathogenesis or progression of TC. Stratification of tumors by histological subtype indicated that TSH increased, preferentially for PTC. The reason for the observed tumour-specific difference in the TSH level is unknown. However, different carcinogenic processes may be involved in the genesis of various well-differentiated thyroid cancers because of the presence of different concentration of serum TSH. As studies for FTC are currently limited, further studies with increasing number of FTC cases are warranted to confirm our findings.

In the diagnostic meta-analysis, we explored the diagnostic performance based on different TSH cut-off values. The areas under SROC were quite similar when different cut-off values were used, while the specificity and sensitivity depended on the TSH cut-off value. However, the overall diagnostic accuracy was poor. No cut-off value can pass the clinical useful test, which indicated TSH alone is not sufficient for confirmation or exclusion of TC. TSH together with other factors may increase the diagnostic accuracy.

TSH stimulates the production and release of thyroid hormones and promotes thyroid cancer growth as well as, invasion, and angiogenesis [70]. Trophic growth effects of TSH in thyroid cancer are well established and are most likely manipulated by TSH receptors on tumor cells [71, 72]. This is consistent with clinical results showing improvement in disease remission and relapse-free survival of DTC patients with TSH suppression [73]. Furthermore, evidence in favour of the TSH receptor's role in thyroid cancer includes the data on autoimmune thyroid disease and thyroid cancer. Although not all studies are in agreement, a meta-analysis of 38 studies showed a 2.8−fold increased incidence of thyroid cancer in patients with Hashimoto's thyroiditis, compared with control population [74]. Recently, genome-wide association studies (GWAS) of levels of TSH in euthyroid individuals has identified associations at 1p36.13 [75], PDE8B [76], and FOXE1 [77, 78]. Several of the newly identified variants confer its risk to TC through serum levels of TSH [77, 78].

Limitations also inevitably existed in this meta-analysis. Firstly, our results were based on unadjusted; while a more precise analysis should be conducted after adjustment for important confounders (e.g., family history, carcinogens exposure and other lifestyle). Secondly, data from the studies exhibit statistically significant heterogeneity. Lack of individual-level data prevents us from making efficient assessment of the sources of heterogeneity. Thirdly, the subgroup analysis of associations between TSH level and tumour size, TNM stage, and lymph node metastasis as well as histological type were performed on the basis of a fraction of all the possible data to be pooled, so selection bias may have occurred and our results may be overinflated. Larger studies with detailed histopathological information are needed to confirm our findings. Fourthly, independence of TSH for TC risk was not investigated in current meta-analysis due to the limit access to individual data. However, many studies included in our-meta-analysis have proved its independence by logistic regression analysis [11, 43]. Finally, differences in the sensitivity and/or specificity of the analytical techniques, or sample degradation during storage, may also contribute to inconsistence between studies.

In conclusion, our meta-analysis of 56 studies indicate that a significant increased serum TSH level were associated with risk of TC. Direct evidence from future prospective study is warranted to clarify a cause-and-effect relationship between TSH and TC as well as its diagnostic value.

## MATERIALS AND METHODS

### Identification and eligibility of relevant studies

We performed this analysis in accordance with the guidelines of the PRISMA (Preferred Reporting Items for Systematic Reviews and Meta-analyses) statement [79]. Epidemiological association studies published before January, 2016, on thyroid stimulating hormone and thyroid cancer were identified by computer-based searches from databases including PubMed, Web of Science, EMBASE, SCOPUS, Cochrane Library databases, CSPD (China Science Periodical Database) and CNKI (China National Knowledge Infrastructure). The search strategy were MeSH terms relating to thyroid stimulating hormone (e.g., “thyroid stimulating hormone”, “TSH”, “thyrotropin”, and “serum thyrotropin level”) in combination with words related to thyroid cancer (e.g., “thyroid cancer”, “thyroid carcinoma”, “thyroid neoplasm”, “thyroid tumor”, and “differentiated thyroid cancer”). No language restriction was applied. The titles and abstracts of potential articles were screened to determine their relevance, and any clearly irrelevant studies were excluded. The full texts of the remaining articles were read to determine whether they contained information on the topic of interest. Furthermore, reference lists of primary studies and review articles were also reviewed by a manual search to identify additional relevant publications.

The outcome of interest was histologically or pathologically confirmed thyroid cancer. Control subjects were defined as thyroid nodule patients or benign surgical patients without thyroid cancer. Eligible studies had to meet all of the following criteria: (1) investigated preoperative serum TSH levels and the risk of TC using either prospective (nested case-control or cohort study) or retrospective design (cross-sectional or retrospective case-control study); (2) original human studies with independent data; (3) no medications that would specifically suppress thyroid function and consequentially TSH response (e.g., methimazole, propylthiouracil or levothyroxine usage); (4) serum TSH were measured separately for cases and controls using a reliable assay; (5) the results were expressed as, or could be estimated into, mean and standard deviation (SD). Major exclusion criteria were: (1) overlapping data, (2) case-only studies and (3) insufficient data.

### Quality assessment and data extraction

For association studies with inconsistent results, the methodological quality should be assessed by appropriate criteria to limit the risk of introducing bias into meta-analyses. A procedure known as ‘Newcastle–Ottawa Scale (NOS)’ has been used to assess the quality of association studies [80]. NOS scores of ≥ 6 were defined as high-quality studies.

Two investigators independently reviewed each eligible article and extracted relevant information. The following data were collected from each study: first author, publication date, diagnostic criterion, study design, age, gender, ethnicity, quantified method of serum TSH, sample size, TNM (tumor node metastasis) stages (I/II, or III/IV, defined according to the AJCC), tumor size (less than 1 cm, 1 or more than 1 cm), lymph node metastasis (yes, or no), histological subtypes, the mean and SD values of serum TSH concentration among cases and controls, the distributions of cases and controls according to different TSH range.. When only the median and range were reported, we used a conversion formula [81] to convert to the mean and SD. We attempted to contact study authors for additional or missing information when needed. Discrepancies in data extraction were resolved by discussion among all authors through consensus.

### Statistical analysis

As serum TSH concentrations were measured using different methods across different studies, a ratio method was used to express the difference in mean TSH between TC patients and controls for each study. In brief, ratio of the mean (RoM) was defined as the mean value of the TC case group divided by that of the control group [82, 83]. Thus, changes in results are expressed as percentages, and their variances are approximated by standard techniques (delta method). To provide quantitative evidence from all studies and maximize statistical power for hypothesis testing, we performed meta-analyses using DerSimonian and Laird's random-effects model which considers both within- and between-study variation to calculate the summary-risk estimate [84]. Heterogeneity across individual studies was calculated using the standard Q-statistic test followed by subsidiary analysis or by random-effect regression models with restricted maximum likelihood estimation [85]. We also calculated the *I*^2^ statistics (*I*^2^ = (Q − df)/Q) to reflect between-study heterogeneity. The percentage of *I*^2^ < 25, 25–50, and > 50 indicates low, moderate, and high heterogeneity, respectively [86]. Sources of heterogeneity were investigated by stratified meta-analyses based on ethnicity, and sample size (No. cases ≥ 500 or < 500). Furthermore, meta-regression analysis was performed to investigate potential sources of heterogeneity by including ethnicity, sample size, age, and sex as covariates. Sensitivity analysis was performed by analysing the influence of each study on the overall estimates and heterogeneity. For diagnostic meta-analysis, we calculated true positives, false positives, false negatives, and true negatives for each individual study that showed frequency of thyroid cancer in accord with serum TSH concentration. The studies were divided into 4 groups based on the cut-off value. (Group1, cut-off values were less than 0.5; Group2, cut-off values were between 0.5 and 1.5; Group3, cut-off values were between 1.5 and 2.5; Group4, cut-off values were larger than 3.5). The diagnostic numbers were used to calculate the pooled sensitivity, specificity, positive likelihood ratio (PLR), negative likelihood ratio (NLR), and corresponding 95% confidence intervals (95% CI) [87, 88]. The PLR is calculated as: sensitivity/(1-specificity) and the NLR is calculated as (1-sensitivity)/specificity. A clinically useful test was defined with a PLR > 10 and a NLR < 0.1. The summary ROC curve (SROC) was generated and the area under the SROC (AUSROC) was calculated [89]. Small study effects was assessed with the funnel plot [90] and Egger's test [91]. All the analyses were done with Stata 10.0 software (STATA Corp., College Station, TX, USA). All P values are two-sided at the *P* = 0.05 level.

## SUPPLEMENTARY MATERIALS FIGURES AND TABLES




