# Supplementary material for: Quantitative assessment of preoperative serum thyrotropin level and thyroid cancer
Source: Oncotarget. 2016 May 6;7(23):34918–29. doi: 10.18632/oncotarget.9201 (PMC5085199; doi:10.18632/oncotarget.9201)
Supplement: Supplementary file 1 [file oncotarget-07-34918-s001.pdf]

Quantitative assessment of preoperative serum thyrotropin level and thyroid cancer

Supplementary Materials

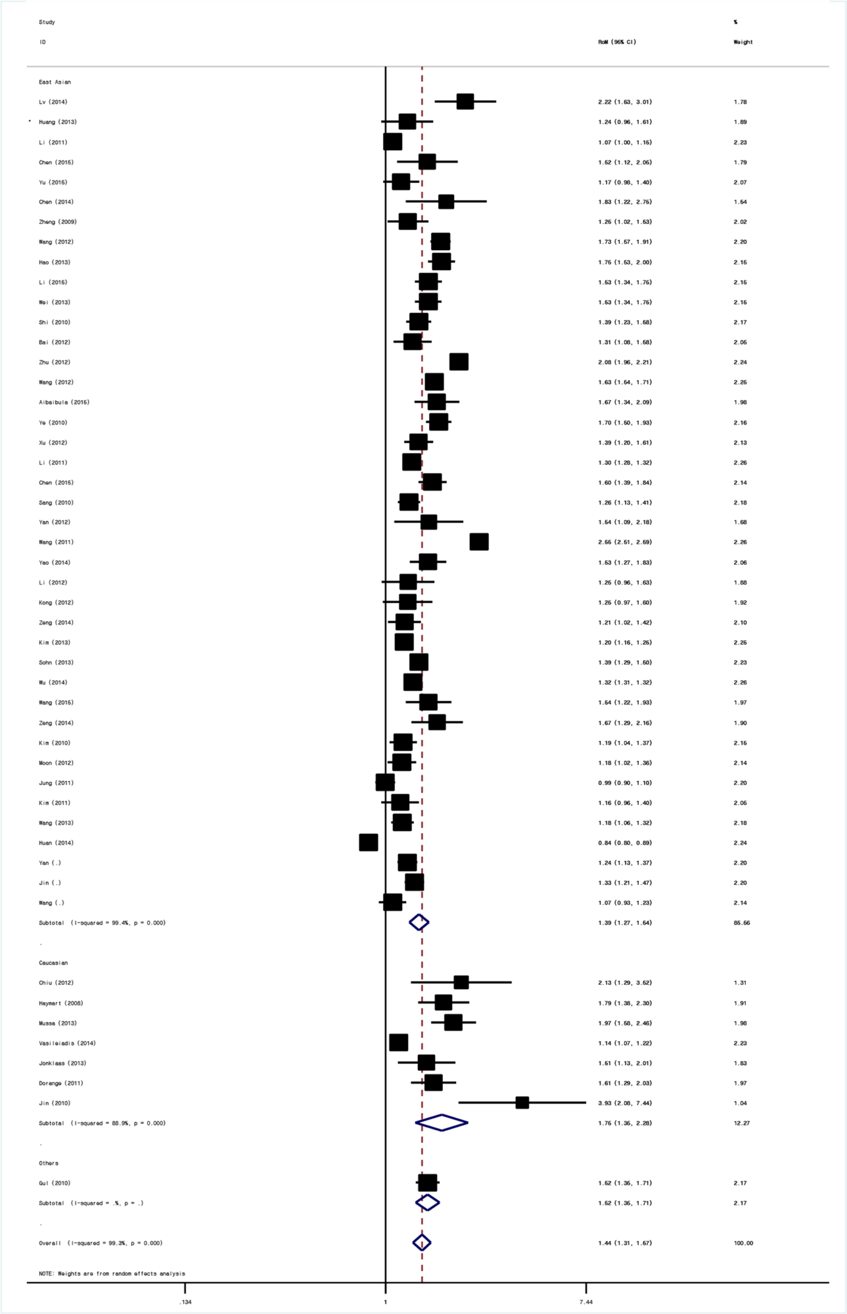

Supplementary Figure S1: Ratio of the mean (RoM) serum TSH level in the thyroid cancer group to the mean TSH level in the control group and 95% CIs without data estimation.

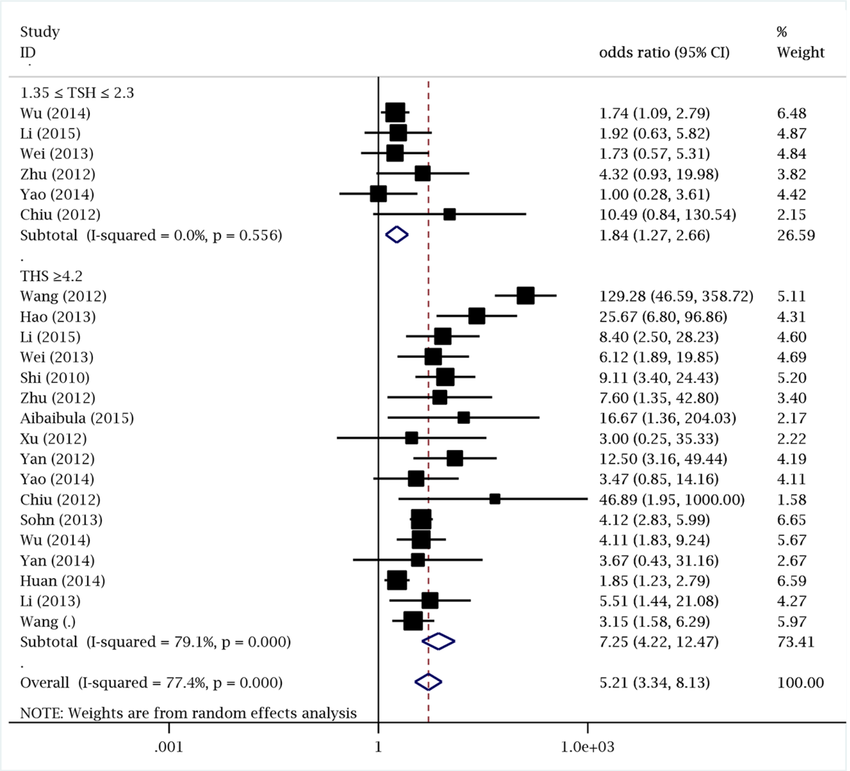

Supplementary Figure S2: Meta-analysis with a random-effects model for the association between thyroid cancer risk and serum TSH level.

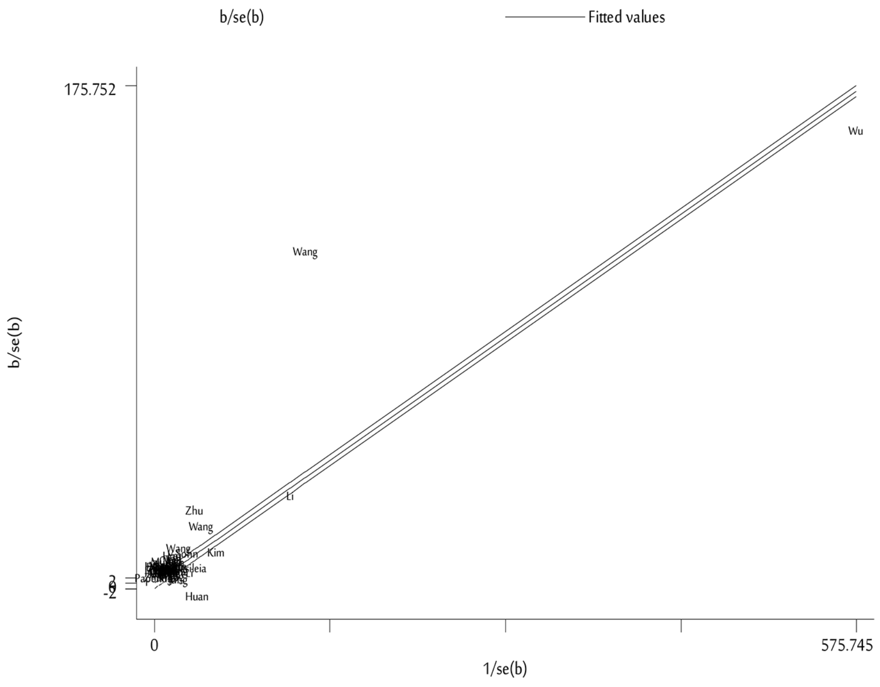

Supplementary Figure S3: Galbraith plot for heterogeneity test of serum TSH level and thyroid cancer.

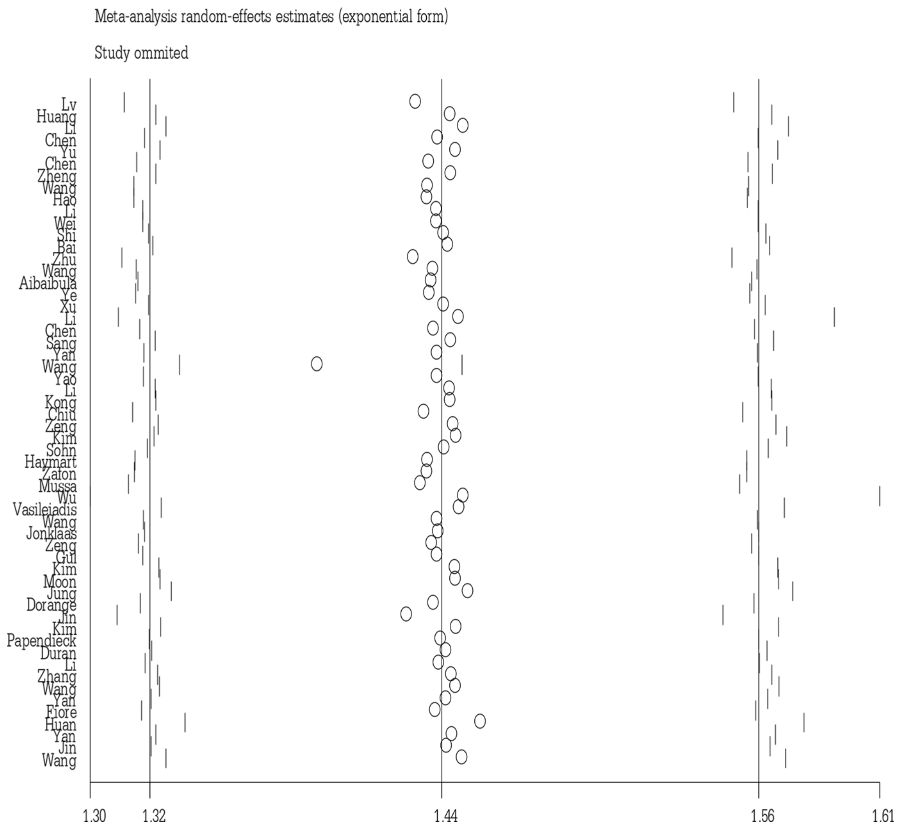

Supplementary Figure S4: Result of sensitivity analyses for serum TSH level and thyroid cancer.

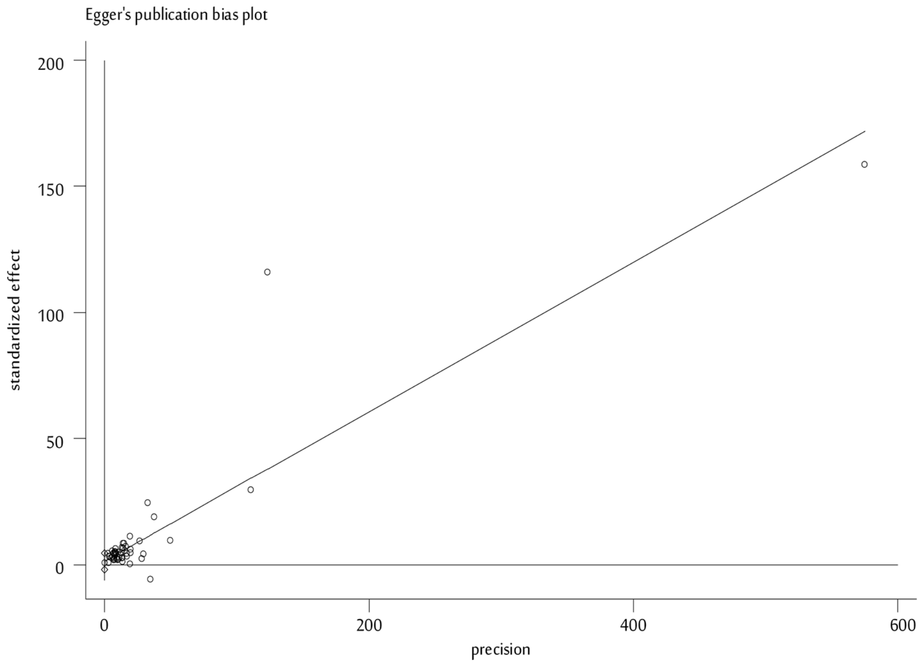

Supplementary Figure S5: Test of small study effect on studies of serum TSH level and thyroid cancer using Egger test.

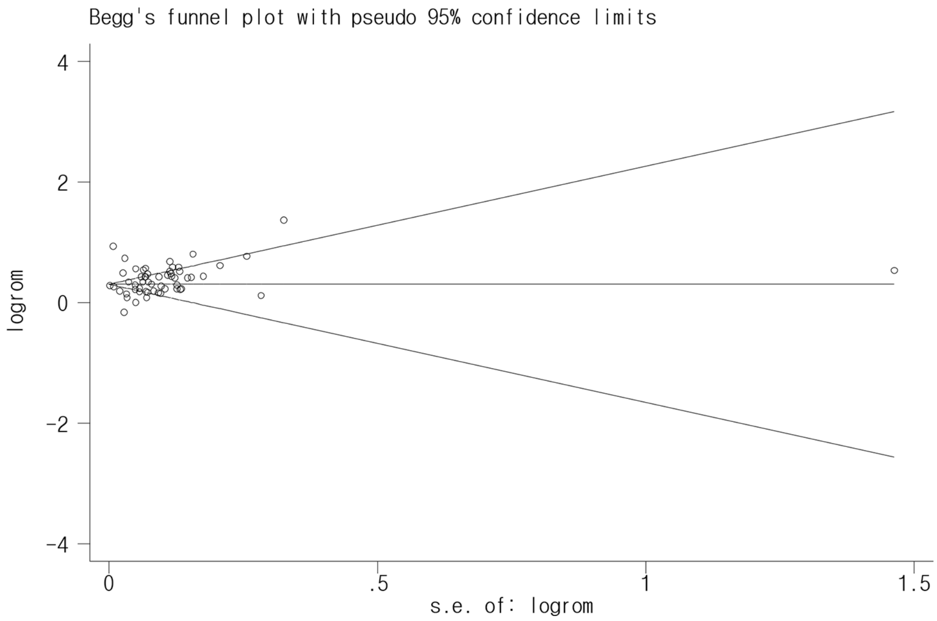

Supplementary Figure S6: Test of small study effect on studies of serum TSH level and thyroid cancer using Begg's funnel plots.

Supplementary Table S1: Main characteristics of studies included in the meta-analysis. See Supplementary_Table_S1
